# Ipsilateral Acetabular and Femoral Neck and Shaft Fractures

**DOI:** 10.1155/2015/351465

**Published:** 2015-06-10

**Authors:** Hideto Irifune, Suguru Hirayama, Nobuyuki Takahashi, Eichi Narimatsu

**Affiliations:** ^1^Department of Emergency Medicine, Advanced Critical Care and Emergency Center, Sapporo Medical University School of Medicine, S-1, W-16, Chuo-ku, Sapporo 060-8543, Japan; ^2^Department of Orthopaedic Surgery, Sapporo Medical University School of Medicine, S-1, W-16, Chuo-ku, Sapporo 060-8543, Japan

## Abstract

Floating hip injuries and ipsilateral femoral neck and shaft fractures are rare. Additionally, the simultaneous occurrence of these injuries is extremely rare, and only one case report of the simultaneous occurrence of these injuries has been published. Here, we report the case of a patient with ipsilateral acetabular and femoral neck and shaft fractures following a suicide attempt. The patient experienced nonunion of the femoral neck and shaft after the initial operation and therefore underwent reconstruction using a femoral head prosthesis with a long stem and interlocking screws. Our procedure may be used in primary and/or secondary reconstruction for ipsilateral acetabular and femoral neck and shaft fractures.

## 1. Introduction

Floating hip injury and ipsilateral femoral neck and shaft fractures are rare and result from high-energy trauma, especially multiple trauma [1–7]. Additionally, the simultaneous occurrence of these injuries is extremely rare, and only one case report of the simultaneous occurrence of these injuries has been published [8].

Here, we report the case of a patient with ipsilateral acetabular and femoral neck and shaft fractures following a suicide attempt.

## 2. Case Presentation

A 38-year-old woman, who had been treated for mental development delay and schizophrenia, was admitted to the intensive care unit of the Department of Traumatology and Critical Care Medicine after a fall during a suicide attempt 30 minutes previously.

She was alert on arrival and her extremities did not have any neurovascular deficits. On physical examination, she was unable to move her right arm and leg, and an approximately 3 cm open wound was noted in the anterolateral thigh region.

**Figure 1:**
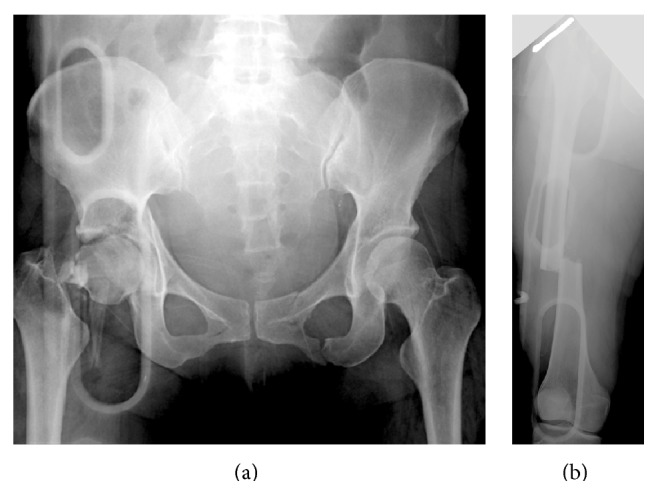
Initial radiographs of the fracture sites. (a) An anteroposterior pelvic radiograph showing an AO type B3 fracture of the right acetabulum and AO type C3 fracture of the right femoral neck. (b) An anteroposterior radiograph showing an AO type A3 fracture of the right femoral shaft with a Gustilo type 2 open wound.

**Figure 2:**
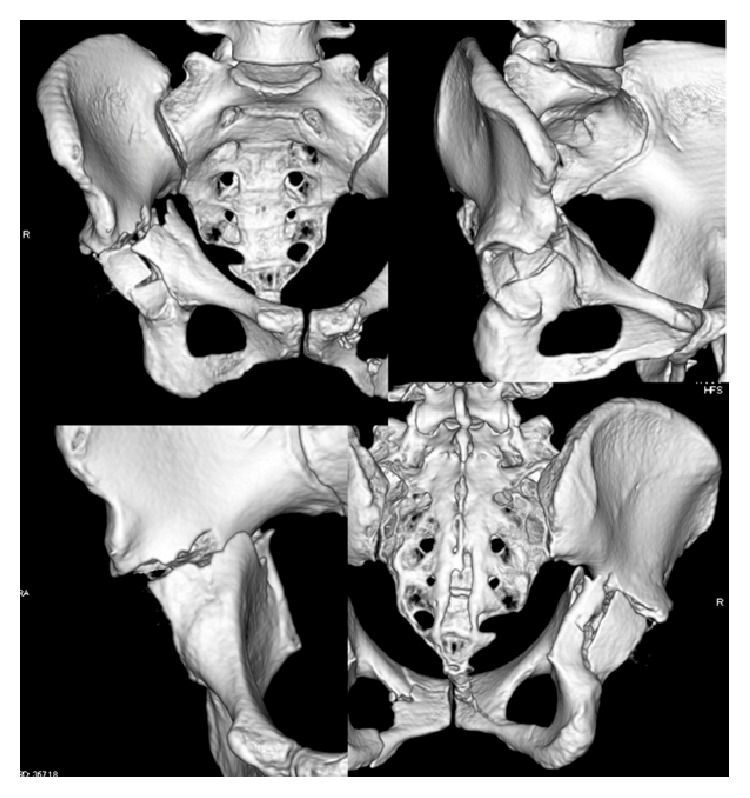
Three-dimensional computed tomography images showing a transverse fracture and posterior wall fracture of the right acetabulum.

Radiographs showed fractured femoral neck and shaft, an acetabular fracture (Figures 1(a) and 1(b)), and a humeral shaft fracture on the right side. Whole body computed tomography showed right pulmonary contusion, liver contusion, and type B3 acetabular fracture, according to the AO classification system (Figure 2). Additionally, her injury severity score was 29.

**Figure 3:**
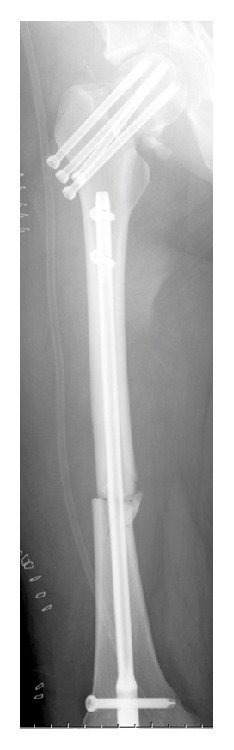
A postoperative radiograph of the femur. Retrograde intramedullary nailing for the right femoral shaft fracture and multiple cannulated cancellous screw fixation for the femoral neck fracture are performed.

She was transferred to the operating room 5 hours after the fall. First, she underwent antegrade intramedullary nailing for the right humerus fracture and retrograde intramedullary nailing for the right femoral shaft fracture simultaneously. Then, she was placed on a traction table and underwent multiple screw fixation with cannulated cancellous screws for the right femoral neck fracture (Figure 3). The operating time was 5 hours, and the intraoperative blood loss was 500 mL.

**Figure 4:**
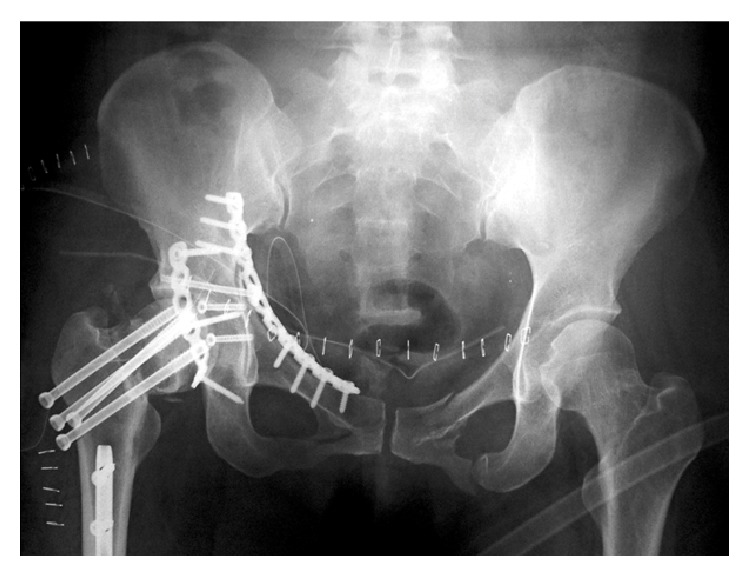
A postoperative radiograph of the acetabulum. Anterior and posterior plate fixation is performed.

Postoperatively, her condition stabilized and she recovered. Seven days after the initial operation, a second operation was performed for the acetabular fracture. First, the transverse fracture was exposed using the modified Stoppa approach and was anatomically reduced and fixed using a reconstruction plate. Then, using the Kocher-Langenbeck approach, posterior wall fixation was performed with a reconstruction plate (Figure 4). The operating time was 5.5 hours and the intraoperative blood loss was 540 mL.

**Figure 5:**
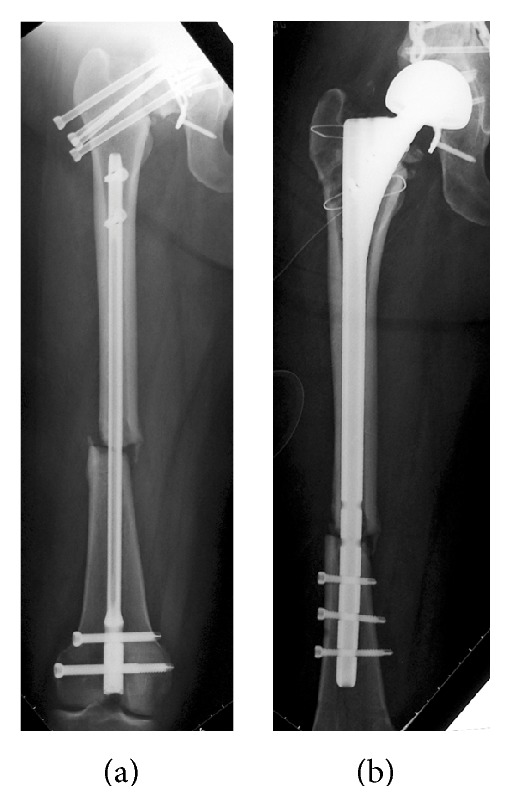
Radiographs of the right femur after the first two operations and after arthroplasty. (a) At 15 weeks after the first two operations, the screws at the femoral neck are loose with nonunion of the femoral neck and shaft. (b) A radiograph showing that arthroplasty was successfully performed.

The early postoperative course was uneventful. Eight weeks after the second operation, partial weight-bearing was allowed. After partial weight-bearing, she complained of right hip and thigh pain. The pain gradually increased, and she was unable to undergo gait training. Radiographs showed hypotrophic nonunion of the right femoral shaft and screw loosening of the right femoral neck (Figure 5(a)). Considering that she had a mental disorder, femoral reconstruction was performed at 15 weeks after the fall.

For femoral reconstruction, we chose a bipolar head prosthesis with a long stem and interlocking screws. First, all implants were removed, and then the long stem prosthesis was inserted, the femoral shaft was dynamized using a large femoral destructor, and locking screws were inserted at the proximal and distal femur.

Postoperatively, she did not complain about right hip and thigh pain and began walking with crutches without weight-bearing on the right leg. Weight-bearing was avoided on the right leg for 4 weeks, and then gradual progression to full weight-bearing was noted at 8 weeks after femoral reconstruction.

**Figure 6:**
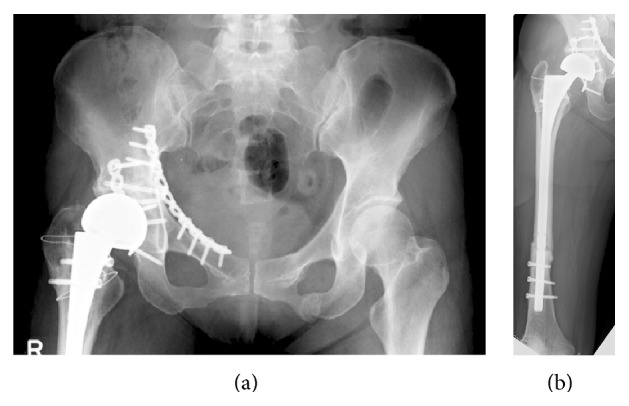
Anteroposterior radiographs of the pelvis (a) and femur (b) 1 year after arthroplasty.

One year after reconstruction, she did not experience any restriction in activities and was very satisfied with the outcome. Additionally, radiographs demonstrated good callus formation in the pelvis and femur, and no loosening of the prosthesis was noted (Figure 6).

## 3. Discussion

Here, we reported the case of a patient with ipsilateral acetabular and femoral neck and shaft fractures following a suicide attempt. The patient was successfully treated with reconstruction using a femoral head prosthesis with a long stem and interlocking screws after nonunion of the femoral neck and shaft following two operations.

Floating hip injury and ipsilateral femoral neck and shaft fractures are rare and result from high-energy trauma, especially multiple trauma [1–7]. Additionally, the simultaneous occurrence of these injuries is extremely rare, and only one case report of the simultaneous occurrence of these injuries has been published [8].

Various treatments have been reported for floating hip injury and ipsilateral femoral neck and shaft fractures. For floating hip injury, Kregor and Templeman stated that the priority of fracture fixation is the acetabular fracture fixation to prevent further damage to the hip joint [4]. However, Liebergall et al. stated that the priority of fracture fixation is the femur because reduction of the acetabulum is easy to perform [1, 2]. For femoral double fractures, Oh et al. reported the use of a retrograde nail for the femoral shaft and screws for the femoral neck [9]. Jain et al. and Watson and Moed used a single antegrade reconstruction nail for shaft and neck fractures [7, 10]. Hung et al. reported the use of plate fixation for shaft fractures and screw fixation for neck fractures [5]. Duygulu et al. reported a case similar to the present case. In their patient, the femoral neck and shaft were fixed using an antegrade reconstruction nail, and then the acetabulum was fixed using plate and screws [8]. In our patient, the femoral shaft was fixed using a retrograde nail, and then the femoral neck was fixed using screws, followed by acetabular reconstruction. We believed that this treatment procedure was the best option for our patient because reduction and fixation could be easily performed.

Unfortunately, our patient experienced nonunion of the femoral neck and shaft after the operation, and early reconstruction was performed because of her mental disorder. A single device is rarely used for femoral reconstruction. However, Oh et al. and Alfonso et al. described the use of a single device for femoral reconstruction [9, 11]. We selected arthroplasty using a femoral head prosthesis with a long stem and interlocking screws for reconstruction. This prosthesis has the features of both a femoral head prosthesis and an intramedullary nail. We obtained good results with the use of this prosthesis for femoral reconstruction, and we believe that this prosthesis may be a primary treatment option for femoral fractures.

In conclusion, simultaneous ipsilateral acetabular and femoral head and shaft fractures are extremely rare, and their treatment may be challenging for orthopedic surgeons and trauma surgeons. For primary ipsilateral femoral neck and shaft fractures, or delayed union and/or nonunion of these fractures, the use of a femoral head prosthesis with a long stem and interlocking screws may be an appropriate reconstruction option.
